# Improving but Inferior Survival in Patients with Chronic Lymphocytic Leukemia in Taiwan: A Population-Based Study, 1990–2004

**DOI:** 10.1371/journal.pone.0062930

**Published:** 2013-04-24

**Authors:** Shang-Ju Wu, Chun-Ju Chiang, Chien-Ting Lin, Hwei-Fang Tien, Mei-Shu Lai

**Affiliations:** 1 Division of Hematology, Department of Internal Medicine, National Taiwan University Hospital, Taipei, Taiwan; 2 Graduate Institute of Clinical Medicine, College of Medicine, National Taiwan University, Taipei, Taiwan; 3 Graduate Institute of Epidemiology and Preventive Medicine, College of Public Health, National Taiwan University, Taipei, Taiwan; 4 Taiwan Cancer Registry, Taipei, Taiwan; Institut national de la santé et de la recherche médicale (INSERM), France

## Abstract

**Background:**

Chronic lymphocytic leukemia (CLL) is much less prevalent in Asian countries. Whether there are differences in survival outcomes between the East and West, however, remain unclear.

**Methods:**

The survival data for CLL patients identified in the Taiwan Cancer Registry database between 1990 and 2004, together with corresponding data in the US Surveillance, Epidemiology, and End Results database, were retrieved. The relative survivals (RS, adjusted for the expected survival in the general population) were estimated in patients diagnosed in three 5-year periods of time.

**Results:**

CLL drastically shortened patients’ life expectancy; more importantly, this negative impact in Taiwan was much larger than that in the US: the 5-year RS in Taiwan and US were 59% and 76%, and the 10-year RS, 45% and 56%, respectively. Nevertheless, survival in Taiwan was better in the periods after 1995 (5-year RS, from 53.0% to 60.6%), a time period corresponding to the introduction of the Taiwan National Health Insurance scheme. Such improvement was largely due to decreased mortality in patients younger than 65 (5-year RS, from 53.5% to 69.1%). Despite the improvement, patients’ RS in Taiwan in recent periods remain steadily 15∼20% inferior to that in the US in both younger and older patient groups.

**Conclusions:**

The improved RS in Taiwan implies that therapeutic advances are changing the prognosis of CLL. The stable RS gap between Taiwanese and the US patients suggests the existence of an ethnic difference in CLL patients’ outcomes.

## Introduction

Chronic lymphocytic leukemia (CLL) is the most common leukemia in adults in Western countries, accounting for 5–11% of non-Hodgkin lymphomas [1], [2], [3]. CLL is much less prevalent in Eastern countries [4], [5], [6]. However, a previous report concerning the epidemiology of CLL in Taiwan revealed a drastic increase in the age-adjusted incidence of CLL, a trend not found in Western countries where the incidence rate of CLL remained steadily stable over time [7]. A strong birth-cohort effect underlying this increasing trend was also identified, suggesting that lifestyle and environmental factors may be involved in the development of CLL in the Taiwanese [7]. Despite this epidemiological difference, it is unknown whether the survival outcomes of CLL are similar between Taiwanese and Western patients. Presently, available data on CLL survival are derived mainly from Western countries [8], [9], [10]. Because CLL is a chronic and indolent disease, a large cohort with a long follow-up period is required to draw conclusions about the survival outcomes. The scarcity of patients in Eastern countries results in that few studies have been able to address the issue in these countries on a large or nationwide scale, or to compare the differences in outcomes between Eastern and Western populations. The aim of the current study is to address this issue by the analyses of population-based survival data in Taiwan and in Western countries to identify possible inter-racial differences in disease outcomes of CLL.

## Patients and Methods

### Data Sources

The Taiwan Cancer Registry (TCR) is a population-based cancer registry that was founded in 1979. It collects information for all newly-diagnosed malignant neoplasms from hospitals with 50 or more beds. The TCR has been steadily improving the quality of database, such as the death certificate only (DCO%) decreased from 18% in 1990–94, 10% in 1995–99 to 3% in 2000–04 [11]. The TCR is estimated to encompass more than 80% of all cancer cases in Taiwan [12], [13], [14], [15].

A total of 1,342 patients aged 15 years or older with a first diagnosis of CLL in the TCR database from 1990 to 2004 were recruited for this study [11]. Cases reported by autopsy only or death certificate only were excluded from the analysis. All Taiwanese patients matched with the death certificate database by personal identification were followed up either to death of the patient or to the end of 2010. Comparable data with the same inclusive criteria were derived from the Surveillance, Epidemiology, and End Results (SEER) public-use database of the National Cancer Institute of the U.S, which includes 18 US cancer registries, between 1990 and 2004 [16]. A total of 25,442 CLL cases from the Caucasian US population were included.

### Statistical Analysis

Relative survival (RS) [17], which expresses the probability of cancer survival after adjustment for competing causes of death, was estimated as the ratio of observed survival to the survival that would have been expected if the cases had been subject only to the age- and sex-specific mortalities observed in the general population. [18] Because of the indolent disease nature and relatively old age of patients, the cause of death in a substantial portion of patients might not be the CLL per se. An important advantage of using RS is that it provides a measure of total excess mortality associated with a diagnosis of CLL irrespective of whether the excess mortality is directly or indirectly related to the cancer, so RS does not rely on the accurate classification of cause of death. As such, RS captures excess mortality because of, for example, the secondary malignancies or underlying cardiovascular diseases, and thus reflects the probability of surviving the cancer of interest rather than the total survival probability. The Ederer II method was used for relative survival analysis using life tables from 1990 to 2004 in the Taiwanese population. [19], [20] To facilitate survival comparisons between countries, age-standardization of relative survival (ASRS) was also done with the international cancer survival standard (ICSS) weights to account for differences in the age distribution of cancer cases. [21] Additionally, the relative excess risk (RER) of RS was estimated using a Poisson model adjusted for age group (15–64 and 65+ years), gender, and time period (1990–1994, 1995–1999, 2000–2004). All analyses, except for the SEER data, were performed using the SAS software.

## Results

### Characteristics of CLL Patients in Taiwan

Table 1 demonstrates the number of CLL cases in Taiwan by age group, gender, and time period. Approximately half of the patients (n = 723, 53.8%) were 65 years or older, and two-thirds (n = 895, 66.7%) were male. Overall, the number of cases increased continuously over time. The male-to-female ratio remained relatively stable over different periods. However, the increase in cases was much more pronounced in the older patient group (from 130 to 332, +155.4% between periods 1990–1994 and 2000–2004) compared to in the younger one (from 141 to 256, +81.5%).

**Table 1:** Number of patients of CLL in Taiwan by age groups, genders, and time periods.

		Period			Total
		1990–94	1995–99	2000–04	
**All**		271(20.2%)	483(35.9%)	588(43.8%)	1342(100%)
**Age**	**15–64**	141(22.8%)	222(35.9%)	256(41.4%)	619(100%)
	**65+**	130(18.0%)	261(36.0%)	332(45.9%)	723(100%)
**Gender**	**Male**	182(20.3%)	333(37.2%)	380(42.5%)	895(100%)
	**Female**	89(20.0%)	150(33.6%)	208(46.5%)	447(100%)

### Much Poorer RS of CLL Patients in Taiwan

**Figure 1:**
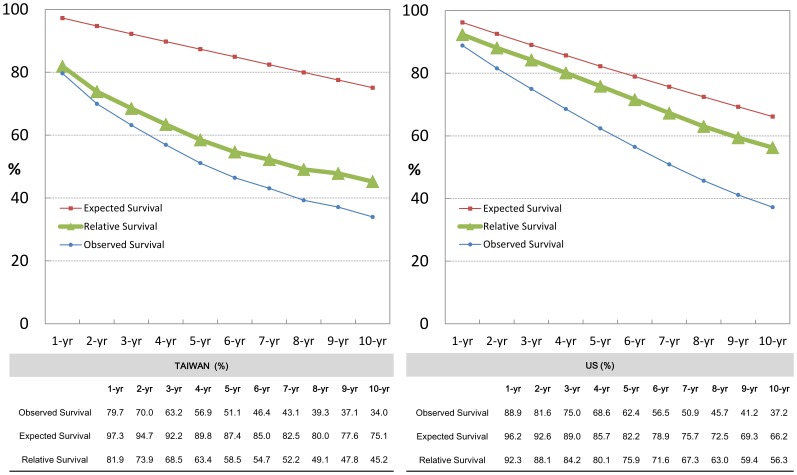
The estimates of observed and relative survivals of patients with CLL among Taiwanese and US patients.

The RS values for Taiwanese and US white patients with CLL are shown in Figure 1. Although the disease is chronic and indolent, the continuously declining RS curves in both series imply that CLL had a substantial negative impact on patients’ survival. Importantly, CLL shortened the life expectancy of Taiwanese patients more dramatically than of US patients. The RS diverged early for Taiwanese patients: it was less than 80% in Taiwan around one year after diagnosis, compared to around four years in the US. The long-term outcomes were also much poorer in Taiwan: the 5-year RS in Taiwan and US were 59% and 76%, and the 10-year RS, 45% and 56%, respectively. The adjusted ASRS data also showed similar results (Supplementary Figure S1A), indicating that the outcome of CLL patients is much worse in Taiwan.

### Better RS in Later Time Periods Among Younger and Male Taiwanese CLL Patients

**Figure 2:**
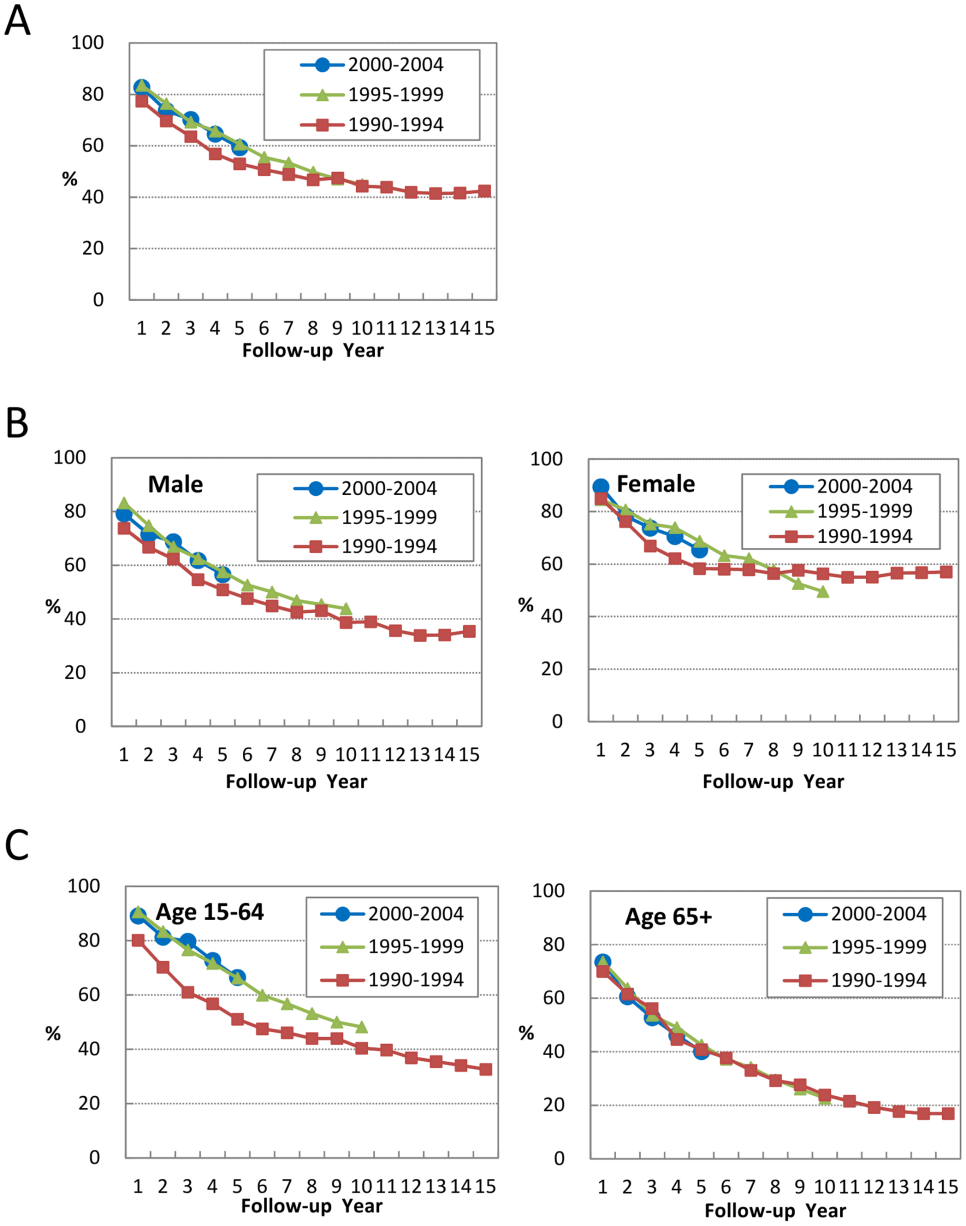
Relative survival curves of patients with CLL in different time periods in Taiwan: (A) for all patients, (B) between genders, and (C) between age groups.

Figure 2A shows an obvious difference in RS between the periods 1990–1994 and the other two periods in Taiwan. In 1990–1994, the 5-year RS of CLL patients was 53.0%, and this proportion increased substantially to 60.3% and 59.0%, respectively, in the later two periods. Figures 2B and 2C show the effects of gender and age group. Generally RS was better in females than in males in every corresponding period. The period effect (after period 1990–1994) on improved RS was also clearly identified in male patients. The improvement of RS, however, was less obvious in female patients; the RS in the later time periods was not steadily higher than during the period 1990–1994, possibly because of the smaller number of cases and the baseline better outcomes. In terms of age groups, RS was not improving in patients aged 65+ years; the RS curves for the three periods overlapped each other. The period effect on survival improvement, however, was drastic in the younger patient group; there was an significant increase in RS after the period 1990–1994.

### Distinctive Period Effect on CLL Survival in Taiwanese Patients

**Figure 3:**
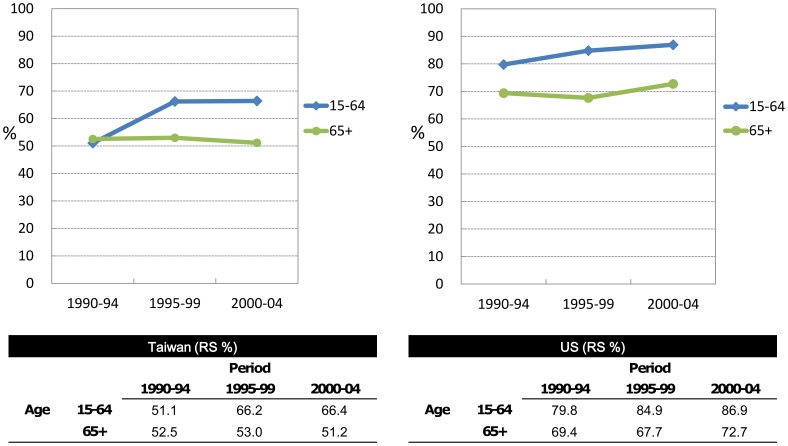
The 5-year relative survival estimates (%) of patients with CLL in different time periods among Taiwanese and the US patients.

Relative survival modeling was performed to identify the individual effects of time period, gender, and age group on RS trends in Taiwan. As shown in Table 2, the statistical test confirmed that period effect did contribute significantly to the RS trends after the adjustment of age and gender effects, a result in agreement with the pattern shown in the previous figures. The estimations of RER were similar between periods 1995–1999 and 2000–2004, a finding also compatible with the patterns shown in the above figures. To determine if this age– group–restricted period effect differed between Taiwan from the US, the period estimates of 5-year RS and ASRS for patients with CLL by age group in corresponding time periods were generated for both Taiwanese and US patients (Figure 3 and Supplementary Figure S1B). The 5-year RS in the US was higher than in Taiwan for every corresponding estimate in both age groups. Interestingly, the trends of 5-year RS among old age groups in both countries were similar, with no obvious changes in the estimates over time. However, the patterns of 5-year RS in younger age groups were different between the two populations; there was a slight but steady improvement in RS over time in the US, whereas there was a drastic improvement in RS in Taiwan in the period 1995–1999. In addition, the differences in RS between younger Taiwanese and younger US patients in the later two time periods were stable; the RS of Taiwanese patients was consistently around 15–20% lower than that of US patients, a gap similar to the differences in older patients between the two countries among all time periods.

**Table 2:** Relative excess risk (RER) of relative survival during the first five years after diagnosis of CLL in Taiwan, 1990–2004.

Parameter		Estimated RER	95% C.I.	p-value
**Period**	2000–2004	0.774	0.606∼0.988	0.039
	1995–1999	0.738	0.573∼0.952	0.019
	1990–1994	1(reference)	.	.
**Gender**	Male	1.407	1.134∼1.746	0.002
	Female	1(reference)	.	.
**Age**	65+	1.615	1.33∼1.962	<0.001
	15–64	1(reference)	.	.

Abbreviations: RER, relative excessive risk; C.I., confidence interval.

## Discussion

The much lower incidences of CLL in Eastern countries are well known. However, the identification of the distinctive incidences of CLL between Taiwan and Caucasian Americans raised the question of whether or not the survival patterns of this chronic, indolent disease also differed ethnically [7]. To our knowledge, the current population-based study represents the first report comparing the survival of CLL patients between Eastern and Western countries with large, nation-wide cancer registry databases to address this issue.

In Taiwan, around half of CLL patients are aged 65 years or older. Overall, the increase in patient numbers over time has been much more drastic than that reported in the US [8]. This observation is compatible with the previous study that reported a distinctive, increasing incidence in Taiwan [7]. In addition, the increase in the case number was greater in the older age group in Taiwan. This phenomenon is different from that in the US, where the numbers of younger patients are increasing more drastically [8]. However, the most obvious increase in incidence in the prior Taiwanese study was also seen in the older age groups [7]. In our prior report the increasing incidence of CLL was likely attributed to the westernization of life styles, which presented as a strong cohort effect, instead of the influence of diagnostic methods, which presented as period effect [7]. The observed pattern of case number changes in the current study is thus in agreement with the incidence trend of CLL in Taiwan.

The most important finding of the current study was the drastic ethnic differences in the outcomes of CLL patients. The survival of CLL patients in Taiwan is remarkably poor; despite the fact that the RS has been better, especially in younger patients, during the recent periods, the improved RS remains much inferior to that of Western patients. Eastern CLL patients have been reported to progress earlier, to have a shorter time-to-first-treatment than Western patients, and to require treatment twice as often as Caucasian patients in the same hospital [22], implying that the outcome difference might be less associated with the availability of medical care or therapeutic medicines but might be more associated with the intrinsic nature of the disease. In the current study, the survival difference between younger Taiwanese and younger US CLL patients in the recent periods is similar to the difference between the older age groups. Such homogeneity of RA gaps also suggests the existence of ethnic differences originating from the intrinsic nature of the disease. The current cancer registry data contains no patients’ profiles about common prognostic factors for CLL, so it is unknown whether Taiwanese patients are more likely to possess poor prognostic features. However, a recent report has identified a new poor prognostic cytogenetic change, trisomy 3, in Taiwanese population [23]. Besides, a recently study identified an East Asian restricted polymorphism of *BIM*, a pro-apoptotic member of the B-cell CLL/lymphoma 2 (BCL2) family critical in cell apoptosis inducing upon tyrosine kinase inhibitor, is strongly associated with the inferior treatment response to tyrosine kinase inhibitors. [24] Interestingly, the same protein has also been addressed to be associated with the treatment resistance of various cytotoxic agents in various cancers. [25], [26], [27], [28] This East Asian restricted *BIM* polymorphism, and other yet indentified genetic polymorphisms, might also lead to poorer treatment responses in East Asian CLL patients and result in the inferior RS in Taiwanese CLL patients found in the current study. These reports imply that Taiwanese patients might have more poor prognostic factors so the disease course might be more aggressive. Further studies focusing on the molecular epidemiology and pathogenesis of CLL will help to clarify this issue.

The drastic improvement in RS in Taiwanese patients in the latter periods is interesting. No equivalent period effect was detected in the US, suggesting that the improvement might have been in response to a specific factor occurring in Taiwan. Several factors might contribute to this kind of period effects, such as the introduction of new techniques or the implementation of new medical care programs. Intriguingly, the Taiwan National Health Insurance, which covers the reimbursement of health care for more than 90% of Taiwan residents, was initiated in 1995 [29], [30], [31]. Most CLL patients in Taiwan are treated in a conservative strategy, i.e. watchful waiting for asymptomatic patients and low-intensity cytotoxic medicines for indicated patients. The National Health Insurance System in Taiwan has made most conventional CLL chemotherapeutic treatments available and achievable to almost all patients as frontline treatment. Novel agents for CLL, including rituximab and fludarabine, have also been available in Taiwan; both novel agents have been reimbursed since 2001 but only for salvage treatments. Better availability of medical care resulting from the Insurance might explain the drastic improvements in outcomes of Taiwanese CLL patients after 1995. The finding further supports the notion that therapeutic advances are changing the prognosis of CLL in Western reports [8], [10].

To further clarify the individual contributions of different biological nature and the treatment differences that might lead to the RS difference between patients in Taiwan and in US, we also retrieved the corresponding data from SEER for Asian/Pacific Islanders who are ethnically closer to Taiwanese but have been exposed to advanced medical care (Supplementary Figure S1A and S1B). We found that, either by the annual trends of ASRS or the 5-year ASRS, the outcomes of CLL patients among Asian/Pacific Islanders living in the United States was just in between that of Caucasian-Americans and patients living in Taiwan in a continuously stable trend, suggesting the influence of both genetic background and the life style. This finding is compatible with our notion that, despite the advances of treatment modalities that have been bettering the outcomes of patients in Taiwan, the persistent inferior RS might suggest the distinctions of genetic backgrounds between the two populations.

Taiwan National Health Insurance has a greater than 90% coverage rate, so older patients should have had similar improvements in medical availability and subsequently better outcomes and RS. However, no such period effect was seen in the older age group. This age-associated discrepancy might be attributed to the current treatment policy for CLL in Taiwan. CLL is a heterogeneous disease and remains incurable with the currently available treatments [32]. Although several novel medicines for CLL have been introduced [33], these medicines all possess substantial toxicities, especially for elderly patients whose tolerance to adverse effects is expected to be much lower. Thus, older patients may not benefit from these newly available therapies because of toxicity concerns. In addition, stem cell transplantation, the only possibly curative modality for CLL, is seldom used in older patients [34], [35]. These factors might mask the improvements associate with the initiation the Taiwan National Health Insurance program in CLL patients in this older age group. Indeed, possibly for the similar reasons, older patients in the US also showed minimal improvements in their survival compared to younger ones [8], [10]. This observation reveals that, although they have substantially improved survival in younger patients, the modern treatment advances have not fulfilled the medical needs of elderly CLL patients.

In the current study, female patients had better RS but a less obvious improvement than male patients. Similar observations have also been reported in Western countries [8], [10]. In prior studies, male CLL patients were associated with more poor-prognostic features, such as more advanced disease stages, unmutated *IgVH* genes, or high CD38 expression [36]. This explains why male patients have poorer survival, but this also explains why male patients may benefit more from improved medical care and emerging novel agents.

A major limitation of the current study was the lack of available data about the treatment modalities and treatment courses of the individual patients because these data are not available in both TCR and SEER. However, RS reflects the net excessive risk of mortality attributable to CLL; improved supportive care is therefore not supposed to contribute to better RS. Thus, although it is not possible to correlate survival changes with any specific treatment modality or medicine, the recent improvement in RS of CLL in Taiwan is likely to be attributable to the better medical care program, which has led to the increased availability of advanced CLL-specific modalities for CLL patients.

In conclusion, in this population-based analysis we have found that the survival of CLL patients in Taiwanese increased dramatically after 1995. Analysis of this trend revealed a period effect contributing to the improved survival of CLL patients in Taiwan, possibly associated with the introduction of the Taiwan National Health Insurance which consequently improves the medical availability, especially for younger patients. This observation implies that therapeutic advances are improving the outcomes of CLL. However, despite the improvement, the overall outcome of CLL is steadily much poorer in Taiwanese patients than in US Caucasians. This distinction in the outcomes, combined with the difference in the incidence trends [7], reflects the existence of ethnic differences in the disease natures of CLL between the East and the West.

## Supporting Information

Figure S1 **The annual trends of ASRS (A) and 5-year ASRS (B) of patients with CLL among Taiwanese, Caucasian Americans and Asian/Pacific Islanders.** (ASRS denotes age-standardized relative survivals).
(DOCX)
