# Supplementary material for: Improving but Inferior Survival in Patients with Chronic Lymphocytic Leukemia in Taiwan: A Population-Based Study, 1990–2004
Source: PLoS One. 2013 Apr 24;8(4):e62930. doi: 10.1371/journal.pone.0062930 (PMC3634739; doi:10.1371/journal.pone.0062930)

**Supplementary Figure S1** The annual trends of ASRS (A) and 5-year ASRS (B) of patients with CLL among Taiwanese, Caucasian Americans and Asian/Pacific Islanders. (ASRS denotes age-standardized relative survivals.)

(A)


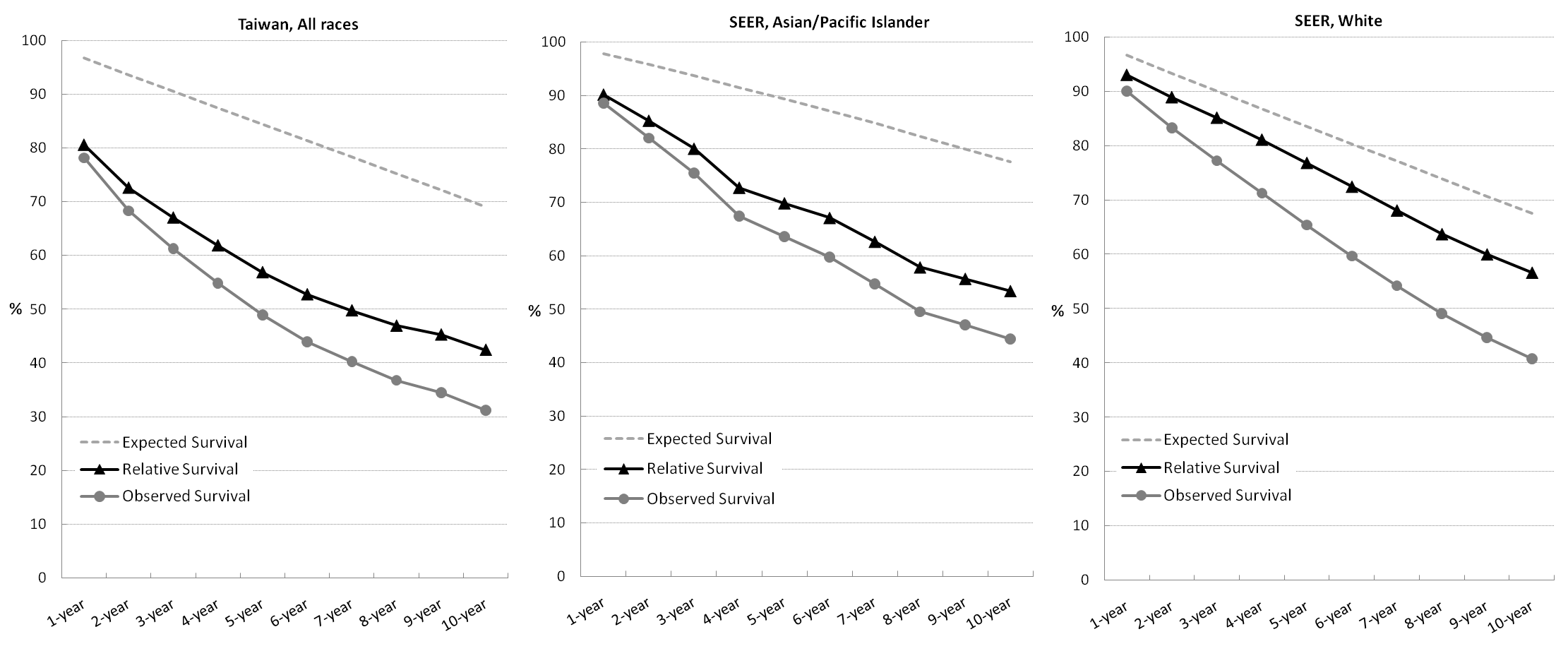


(B)


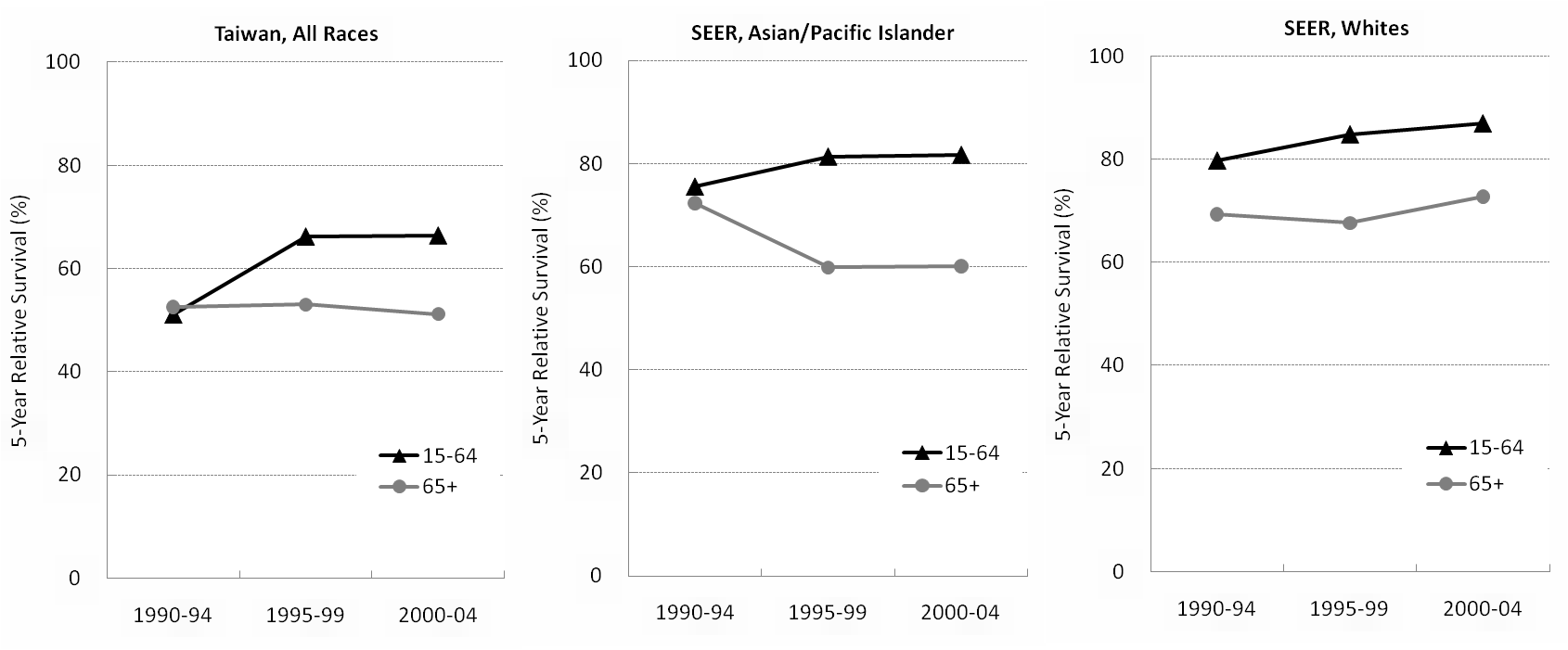

Supplement: Figure S1 — The annual trends of ASRS (A) and 5-year ASRS (B) of patients with CLL among Taiwanese, Caucasian Americans and Asian/Pacific Islanders. (ASRS denotes age-standardized relative survivals). (DOCX) [file pone.0062930.s001.docx]
